# SunGold Kiwifruit Supplementation of Individuals with Prediabetes Alters Gut Microbiota and Improves Vitamin C Status, Anthropometric and Clinical Markers

**DOI:** 10.3390/nu10070895

**Published:** 2018-07-12

**Authors:** Renée Wilson, Jinny Willis, Richard B. Gearry, Alan Hughes, Blair Lawley, Paula Skidmore, Chris Frampton, Elizabeth Fleming, Angie Anderson, Lizzie Jones, Gerald W. Tannock, Anitra C. Carr

**Affiliations:** 1 Department of Medicine, University of Otago, Christchurch 8140, New Zealand; renee.wilson@postgrad.otago.ac.nz (R.W.); richard.gearry@cdhb.health.nz (R.B.G.); chris.frampton@otago.ac.nz (C.F.); 2 New Zealand Nursing Organisation, Christchurch 8140, New Zealand; jinny.willis@nzno.org.nz; 3 Microbiome Otago, University of Otago, Dunedin 9054, New Zealand; gerald.tannock@otago.ac.nz; 4 Department of Microbiology and Immunology, University of Otago, Dunedin 9054, New Zealand; alan.hughes@otago.ac.nz (A.H.); blair.lawley@otago.ac.nz (B.L.); 5 Department of Human Nutrition, University of Otago, Dunedin 9054, New Zealand; paula.skidmore@otago.ac.nz (P.S.); liz.fleming@otago.ac.nz (E.F.); angie.anderson@otago.ac.nz (A.A.); lizzieig71@gmail.com (L.J.); 6 Riddet Centre of Research Excellence, Massey University, Palmerston North 4442, New Zealand; 7 Department of Pathology & Biomedical Science, University of Otago, Christchurch 8140, New Zealand

**Keywords:** vitamin C, blood pressure, waist circumference, glucose, glycaemic control, HbA1c, kiwifruit, gut microbiota, *Coriobacteriaceae*

## Abstract

Kiwifruit are a nutrient dense food and an excellent source of vitamin C. Supplementation of the diet with kiwifruit enhances plasma vitamin C status and epidemiological studies have shown an association between vitamin C status and reduced insulin resistance and improved blood glucose control. In vitro experiments suggest that eating kiwifruit might induce changes to microbiota composition and function; however, human studies to confirm these findings are lacking. The aim of this study was to investigate the effect of consuming two SunGold kiwifruit per day over 12 weeks on vitamin C status, clinical and anthropometric measures and faecal microbiota composition in people with prediabetes. This pilot intervention trial compared baseline measurements with those following the intervention. Participants completed a physical activity questionnaire and a three-day estimated food diary at baseline and on completion of the trial. Venous blood samples were collected at each study visit (baseline, 6, 12 weeks) for determination of glycaemic indices, plasma vitamin C concentrations, hormones, lipid profiles and high-sensitivity C-reactive protein. Participants provided a faecal sample at each study visit. DNA was extracted from the faecal samples and a region of the 16S ribosomal RNA gene was amplified and sequenced to determine faecal microbiota composition. When week 12 measures were compared to baseline, results showed a significant increase in plasma vitamin C (14 µmol/L, *p* < 0.001). There was a significant reduction in both diastolic (4 mmHg, *p* = 0.029) and systolic (6 mmHg, *p* = 0.003) blood pressure and a significant reduction in waist circumference (3.1 cm, *p* = 0.001) and waist-to-hip ratio (0.01, *p* = 0.032). Results also showed a decrease in HbA1c (1 mmol/mol, *p* = 0.005) and an increase in fasting glucose (0.1 mmol/L, *p* = 0.046), however, these changes were small and were not clinically significant. Analysis of faecal microbiota composition showed an increase in the relative abundance of as yet uncultivated and therefore uncharacterised members of the bacterial family *Coriobacteriaceae*. Novel bacteriological investigations of *Coriobacteriaceae* are required to explain their functional relationship to kiwifruit polysaccharides and polyphenols.

## 1. Introduction

Diabetes is one of the largest global health crises, affecting 415 million adults worldwide, with type 2 diabetes mellitus (T2DM) accounting for at least 90% of all cases of diabetes [1]. People with diabetes are at a higher risk of developing a number of disabling and life-threatening health conditions [1]. Prediabetes reflects a stage between normal glucose tolerance (NGT) and T2DM. Those with prediabetes are at high risk of progressing to T2DM, although this is not inevitable [2]. In routine practice, prediabetes is characterized by an increase in HbA1c. Results from the 2008/2009 New Zealand Adult Nutrition Survey (NZANS) provided data on the prevalence of diabetes and prediabetes using the American Diabetes Association (ADA) criteria and found that the prevalence of diabetes and prediabetes in New Zealand was 7.0% and 25.5% respectively [3]. The alarmingly high prevalence of people with prediabetes necessitates further research to explore modifiable causative factors for prediabetes.

Hyperglycaemia (elevated blood glucose concentrations) and insulin resistance are implicated in the pathogenesis of micro- and macrovascular complications [4]. Maintaining normal blood glucose levels through diet is one of the main goals in the management of a glucose metabolism disorder (diabetes or prediabetes) [5]. Higher plasma vitamin C is associated with reduced insulin resistance and improved blood glucose control [6,7,8,9,10]. Since hyperglycaemia is associated with increased oxidative stress, a role for antioxidants such as vitamin C in the prevention of T2DM and/or the reduction of complications is a reasonable proposition. However, there have been mixed findings reported in randomised controlled trials (RCTs) of supplementation with vitamin C on glycaemic control and insulin sensitivity [11,12,13]. A recent meta-analysis of 15 RCTs investigating vitamin C supplementation and insulin resistance and biomarkers of glycaemic control (fasting glucose, HbA1c) found that doses of ≥200 mg/day vitamin C significantly reduced glucose concentrations in patients with T2DM, particularly if the intervention was for more than 30 days and in older individuals [14]. Furthermore, a recent 12 month RCT found that treating those with T2DM with both metformin and vitamin C was more effective at reducing HbA1c and risk factors for diabetes-related long-term complications than treating with metformin alone [15].

SunGold kiwifruit are one of the best dietary sources of vitamin C (160 mg/100 g) among fruit and vegetables [16]. Regular consumption of two SunGold kiwifruit has been shown to significantly increase the plasma vitamin C levels in men with inadequate levels of plasma vitamin C [17]. Virtually all fruits represent a source of sugars and therefore moderate consumption is recommended in people with T2DM. However, there are differing amounts of sugar and other nutrients in various fruit and portion sizes vary markedly. Kiwifruit, eaten as a whole fruit, have a low glycaemic impact and are, therefore, thought to be a suitable choice for those with prediabetes and T2DM [18].

In addition to having a positive effect on glycaemic control, gold kiwifruit contain dietary fibre (1.4 g/100 g in raw Zespri SunGold kiwifruit) [16] and polyphenols which resist digestion by human enzymes and are degraded by bacteria in the digestive tract, stimulating the growth or activity of certain bacteria in the colon. The fibre in kiwifruit comprises both soluble (e.g., pectin) and insoluble (e.g., hemicelluloses and celluloses) components which make up cell walls [19].

T2DM has been reported to be associated with an alteration in the usual balance of bowel bacterial species, described as a bacterial dysbiosis [20]. Differences in the gut microbiota have been demonstrated between individuals with T2DM and healthy individuals in previous research [21,22]. Further, the finding of differences in gut microbiota between healthy individuals, individuals with prediabetes and those with T2DM suggests a potential association between gut ecology and the progression from normal glucose tolerance to T2DM [23]. A logical extension is the hypothesis that manipulation, by dietary change, of the relative abundances of particular bacterial taxa within the gut microbiota could play a role in managing T2DM [24].

On the basis of in vitro experiments, SunGold Kiwifruit consumption has been speculated to affect the composition and functioning of the bowel microbiota [25]. However, the outcomes of this study need to be confirmed in human intervention studies. Therefore, the aim of the present study was to determine whether gold kiwifruit consumption by a prediabetic cohort altered gut microbiota composition, increased plasma vitamin C concentrations and improved glycaemic control or prevented further deterioration of glucose intolerance.

## 2. Materials and Methods 

### 2.1. Study Participants

The study was approved by the Southern Health and Disability Ethics Committee (consent no. 16/STH/87) and was included in the Australian New Zealand Clinical Trials Registry (ACTRN12616000858493). Written informed consent was obtained from all participants. Individuals ≥18 years meeting the inclusion criteria detailed below were recruited by a range of methods including: advertisements in local newspapers, flyers in general practice and other primary care health settings, e-mailing staff at local large businesses including the Canterbury District Health Board (CDHB), participants from previous studies were contacted, study information was posted to those who had attended prediabetes education classes and flyers placed at local businesses in Christchurch, New Zealand.

A total of 41 individuals underwent a screening questionnaire and venous blood test to measure HbA1c to ascertain eligibility for the study. Twenty-six participants were enrolled and 24 participants completed the study. Four participants (one of whom did not complete the trial) were excluded from the gut microbiota analyses due to starting a course of antibiotics during the trial. One patient left the study due to a family bereavement. Another participant was admitted to hospital for a pre-existing condition and had to conclude involvement in the study.

#### 2.1.1. Inclusion Criteria

Participants (≥18 years) who met the ADA diagnostic criteria for prediabetes (HbA1c result of 39–46 mmol/mol) at baseline were recruited. HbA1c is routinely used as the diagnostic test for diabetes in New Zealand [26]. The red blood cell has a 90–120 day lifespan and during this time haemoglobin is glycated in proportion to the mean exposure to glucose [26]. Accordingly, a three-month intervention period was chosen for this trial.

#### 2.1.2. Exclusion Criteria

Individuals unable to give informed consent, those with an HbA1c outside the diagnostic range for prediabetes (HbA1c result of 39–46 mmol/mol), those with a previous diagnosis of diabetes, or those on diabetes medications such as Metformin. In addition, individuals who had taken antibiotics in the last month, those with a medical history of significant gastrointestinal disease (for example, inflammatory bowel disease), previous bowel resection, those with a known kiwifruit allergy, women who were pregnant, breastfeeding or planning a pregnancy and those planning to travel overseas in the three months post selection (trial period) were also excluded.

### 2.2. Study Design

This was a pilot intervention trial where baseline measurements were used as the control measures for comparison. Participants were screened with a questionnaire and venous blood test to measure HbA1c. If eligible they started a lead in phase where they were asked to not eat any kiwifruit (green or gold) for seven days. During this time, they also completed an estimated food diary by writing down everything they ate or drank over three non-consecutive days (two weekdays and one weekend day) to analyse their usual dietary intake.

After the lead-in phase, Zespri SunGold kiwifruit (Gold3, *Actinidia chinensis*) were delivered to participants weekly. Participants were provided with twice as many fruit as were required and were advised that extra fruit could be shared with family and friends but to ensure that they had enough to last them until the next delivery. The participants were asked to store the fruit in a refrigerator or cool place. The kiwifruit were selected to be of the same size and quality. The average weight of one SunGold kiwifruit was 132 g (whole fruit) and 95 g for the flesh (28% accounts for the skin, [16]). Participants were asked to consume the flesh of two SunGold kiwifruit every day for twelve weeks. Strategies to avoid forgetting the fruit were discussed such as eating them at the same time every day and having two kiwifruit out on the bench every day so the participants knew whether or not they had eaten them. Participants were asked not to eat any kiwifruit other that the two study fruit during the 12 weeks. As the study was a free-living situation, participants were asked to consume the fruit as part of their usual diet. Participants were asked not to eat the skin and to eat the flesh raw and not crushed or blended in a smoothie. Participants were asked to maintain their normal dietary and lifestyle habits for the duration of the trial.

**Figure 1:**
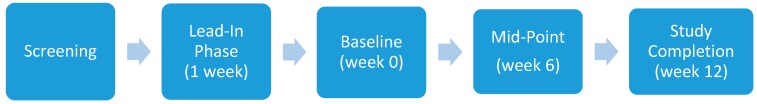
Study timeline.

Following screening and the lead-in week there were three study visits; baseline, week six and week twelve (Figure 1). Table 1 shows the information that was collected at each study visit.

### 2.3. Anthropometric Measures

#### 2.3.1. Weight (kg)

Participants were asked to remove their footwear and heavy outer clothing such as jackets and were weighed to the nearest 0.1 kg on calibrated Tanita scales (Model BWB-800A, Tanita Corporation, Tokyo, Japan).

#### 2.3.2. Height (m)

Measured once at baseline to the nearest mm using calibrated height measures.

#### 2.3.3. BMI (kg/m^2^)

Calculated by weight in kilograms divided by height in metres squared.

#### 2.3.4. Waist Circumference (cm)

The WHO STEPwise Approach to Surveillance protocol for measuring waist circumference was used. The measurement was made at the approximate midpoint between the lower margin of the last palpable rib and the top of the iliac crest [27]. The tightness of the tape was controlled by using a Gulick II Measuring tape (Model 67020, Country Technology Inc., Gays Mills, WI, USA). Three measurements were taken at each study visit. The measurements for each participant were averaged. The coefficient of variation associated with the measurement error for waist circumference was 0.65%.

#### 2.3.5. Hip Circumference (cm)

Measured to the nearest mm around the widest portion of the buttocks with the tape parallel to the floor using a Gulick II Measuring tape, as described above. Three measurements were taken at each study visit. The measurements for each participant were averaged. The coefficient of variation associated with the measurement error for hip circumference was 1.86%.

#### 2.3.6. Waist-To-Hip Ratio

Calculated by dividing the waist circumference by the hip measurement.

#### 2.3.7. Fat Mass (%)

Measured using the BIA 450 Bioimpedance Analyser (Biodynamics Corporation, Seattle, WI, USA). Patient assessments were conducted using a connection between the individual’s wrist and ankle and the analyser, using standard ECG sensor pad electrodes (CONMED Corporation, Utica, NY, USA). One participant had a pacemaker so was excluded from this measure.

#### 2.3.8. Blood Pressure (mmHg)

Measured using an automated blood pressure monitor (Bp TRU, BTM-300, Omron Healthcare Co., Ltd., Muko, Kyoto, Japan). Three measurements were taken at each study visit. The measurements for each participant were averaged. The coefficient of variation associated with the measurement error for systolic and diastolic blood pressure were 2.99% and 5.16% respectively.

### 2.4. Blood Parameters

Venous blood samples were collected after a 12-hour fast and were analysed for fasting glucose as an additional measure of glycaemic control. EDTA plasma was collected and immediately stored on ice and centrifuged for 10 minutes at 1000× *g* (2500 rpm) at 4 °C. The plasma was removed and immediately frozen at −80 °C for batch analysis of vitamin C.

#### 2.4.1. Glucose

Fasting glucose was measured in blood collected in fluoride oxalate venoject tubes by standard methods (Glucose Hexokinase Enzymatic Assay, Abbott c series analyser, Abbott Park, IL, USA) at an IANZ laboratory. The coefficient of variation associated with the measurement of glucose in plasma is 1.5% at 6.78 mmol/L [28].

#### 2.4.2. Vitamin C

Frozen plasma samples were rapidly defrosted, acidified and stabilised with perchloric acid and a metal chelator. The supernatants were then treated with a reducing agent prior to analysis to recover any vitamin C that had become oxidised during handling or storage [29]. The samples were analysed by the gold standard method of high performance liquid chromatography with electrochemical detection as described previously [17,29].

#### 2.4.3. HbA1c

Determined in EDTA blood by standard methods (Bio-rad Variant HPLC, Bio-Rad, Hercules, CA, USA) at an IANZ laboratory. The coefficient of variation associated with the measurement of HbA1c at the IANZ is 0.66% at HbA1c of 38 mmol/mol [30].

#### 2.4.4. Lipid Parameters

Total cholesterol, HDL-cholesterol, LDL-cholesterol and triglycerides were determined in lithium heparin blood by standard methods (Abbott c series analyser, Abbott Park, IL, USA) at an IANZ laboratory.

#### 2.4.5. hs-CRP

The inflammatory marker hs-CRP was measured using end-point nephelometry at an IANZ laboratory.

#### 2.4.6. Hormones

EDTA plasma was collected and centrifuged for 10 minutes at 1000× *g* (2500 rpm) at 4 °C. The plasma was frozen at −80 °C and stored for batched analyses. Ghrelin, leptin and adiponectin were determined by the Christchurch Heart Institute, Department of Medicine, University of Otago, Christchurch.

Ghrelin was measured by an in-house RIA following extraction from plasma using Sep Pak C18 cartridges, as described previously [31]. The assay recognises the total circulating ghrelin (i.e., both octanoyl and non-octanoyl forms). The cross reactivities of other peptides in the assay, including vasointestinal peptide, prolactin, galanin, growth hormone releasing hormone, neuropeptide Y, brain natriuretic peptide, atrial natriuretic peptide, endothelin-1 and angiotensin II were all less than 0.03%. The RIA had a mean detection limit of 10.8 ± 0.8 pmol/L and mean ED50 of 136.2 ± 10.0 pmol/L over 23 consecutive assays.

Leptin and adiponectin were measured using commercial ELISA from BioVendor (Brno, Czech Republic), Research and Diagnostic products (RD191001100 Human Leptin ELISA and RD191023100 Human Adiponectin ELISA) according to the manufacturer’s instructions.

Insulin was measured using the Roche Cobas e411 method in an IANZ laboratory. After storage at −80 °C, thawed plasma was pre-treated using 25% polyethylene glycol to precipitate antibodies.

### 2.5. Faecal Microbiota Analysis

Participants provided a faecal sample at their study appointment, which they had collected at home in a sterile collection bottle no more than 24 h prior to their study visit. The sample was either stored in an insulated bag with an ice pack or in a home refrigerator. Samples were transported in a coolie bag with an ice pack to their study appointment. All samples were processed within 24 h of the patient collecting their sample. The samples were processed in a microbiological sterile hood into four aliquots of 0.5–1.0 g of faeces and stored in sterile 1.5 mL Eppendorf tubes at −80 °C for batched analyses. Faecal sample collection methodology was adapted from the International Human Microbiome standard operating procedures and with the Human Microbiome Project Methodology (www.ncbi.nlm.nih.gov/projects/gap/cgi-bin/document.cgi?study_id=phs000228.v3.p1&phd=3190#sec114).

#### 2.5.1. DNA Extraction and Sequence Analysis

DNA was extracted from stool using the MoBio PowerSoil DNA Isolation kit according to the Human Microbiome Project standard operating procedures (www.ncbi.nlm.nih.gov/projects/gap/cgi-bin/document.cgi?study_id=phs000228.v3.p1&phd=3190#sec114). Genomic DNA was submitted to Argonne National Laboratories for barcoded amplification of the V4 region of the bacterial 16S rRNA gene and sequencing on a MiSeq (Illumina, San Diego, CA, USA) instrument. DNA sequences were processed using the QIIME v. 1.9.1 and vsearch v.2.0.2 suite of programs [32,33]. Genus level taxonomy was obtained by filtering Operational Taxonomic Unit (OTU) tables containing taxonomic data generated using the RDP classifier, extracting representative sequences and using BLAST to identify genus level matches, where possible, within the NCBI database. Alpha- and beta-diversity analysis of phylogenetic data compared the coverage and richness (number of phylotypes) and similarity or difference in the composition of the stool microbiota between subjects and treatment groups.

#### 2.5.2. Faecal Water Content

Water content of faeces reflects gut transit time. Shorter transit time means more water in faeces. Shorter transit time means less time for bacterial replication and might favour species with shorter doubling times. For each faecal sample, approximately 200 mg of faeces was placed in a pre-weighed microfuge tube, the weight was recorded and the tube with cap open was placed in a 37 °C incubator. The tubes were dried until a constant dry weight was obtained and percentage water content was then calculated.

### 2.6. Questionnaires

#### 2.6.1. Demographic Information

Participants recorded their date of birth, sex, ethnicity and qualification. Information on medical history, alcohol consumption and smoking status was also collected.

#### 2.6.2. Medication and Supplement Use

At baseline participants were asked to record any medications (prescription or non-prescription) they were currently taking, the dose, the number per day and the length of time they had been taking the medication. They were also asked if they were taking any dietary supplements, how frequently they took the supplement and when their last dose was. At weeks 6 and 12 they were asked if they had stopped taking any medications/supplements or if they had started taking a new medication or supplement and if so the details were recorded.

#### 2.6.3. Dietary Intake

Participants completed a three day (two weekdays and one weekend day) estimated food diary during their lead-in week and during their final week of consuming the SunGold kiwifruit (week 12). Participants’ daily dietary intake was calculated as an average over the three days. Participants were asked to record everything they ate or drank over the three days; describing each item in detail, including cooking details and any salt, sugar, spices and sauces they may have added before eating. They were also asked to record the brand name of each food, drink or cooking ingredient and also if possible to attach the wrappers of foods to provide the nutrition information. Tips were provided to assist with estimation of portion sizes such as using household measures, for example, two rounded teaspoons of sugar. A book with photos of commonly eaten foods of different portion sizes was also provided. Participants were also encouraged to take photos of their meals in addition to completing the food diary with estimated portions. Once completed the diary was reviewed during their study appointment to add any missing information if necessary.

The food diaries were entered into Kaiculator (version 1.08d), a nutrient analysing programme developed by the Department of Human Nutrition at the University of Otago, New Zealand. Kaiculator uses the 2014 version of the New Zealand food composition database, NZ FOODfiles. The methodology for entering the food diaries has been described for our previous observational study [34]. Average total daily energy and fibre were calculated along with percent energy values for total fat, carbohydrate and protein. Dietary vitamin C was also analysed. Food groups for fresh fruit and total fruit were created and calculated for comparison of fruit intake prior to (lead-in phase) and when consuming the two SunGold kiwifruit daily (week 12). At week 12 both fresh and total fruit, with and without the SunGold kiwifruit, were created for comparison with baseline fruit intake. Kiwifruit consumption recorded in the week 12 food diary was used as an indication of compliance.

Percent energy from macronutrients per day was calculated from the average total daily dietary intake as follows [35]:

- percent energy from fat = (fat (g/day) × 37.7 kJ/g)/energy (kJ/day)
- percent energy from carbohydrate = (carbohydrate (g/day) × 16.7 kJ/g)/energy (kJ/day)
- percent energy from protein = (protein (g/day) × 16.7 kJ/g)/energy (kJ/day)

#### 2.6.4. Physical Activity

A physical activity questionnaire was collected to monitor that participants maintained their usual activity levels. Participants completed the self-administered International Physical Activity Questionnaire (IPAQ) short form. The questionnaire asks about physical activity over the previous seven days. Participants were scored as MET-minutes per week using the IPAQ scoring protocol [36].

### 2.7. Statistical Analysis

Standard descriptive statistics including means, medians, standard deviations, interquartile ranges, frequencies and percentages were used to describe the baseline demographic, anthropometric, laboratory and questionnaire data. The changes in these measures over time were compared by paired t-tests and Wilcoxon Signed rank tests as appropriate. Changes in taxonomic relative abundance (RA) and diversity data was statistically analysed using Wilcoxon signed rank tests and are presented as medians and interquartile ranges. The association between vitamin C concentrations and intakes and demographic, dietary, laboratory and anthropometric measures were tested by Pearson’s correlation coefficients. Changes in anthropometric and laboratory measures were compared between the two vitamin C adequacy groups by one-way ANOVA. A two-tailed *p* < 0.05 was taken to indicate statistical significance.

## 3. Results

The average age of the participants was 66 years and ranged from 44 to 85 years old. There was an equal number of male and female participants and the majority of participants were European (81%) (Table 2). There was a mix of educational qualifications, which is expected given the age range of the participants. There were fewer participants currently smoking (15%) compared to the number of participants who reported being ex- (39%) or non-smokers (46%). The majority of participants reported consuming alcohol (73%) (Table 2).

### 3.1. Dietary Intakes: Macronutrients, Micronutrients, Fruit Intake

There were no significant differences in daily energy, fibre, protein, total fat and total available carbohydrate when week 12 was compared to baseline (Table 3). There was a non-significant trend towards an increase in total available sugars and when investigated further results showed the daily intake of fructose and glucose to be significantly higher at week 12 compared to baseline (*p* < 0.001) and the intake of sucrose to be significantly lower at week 12 compared to baseline (*p* = 0.018). Starch intake was not significantly different at week 12 compared to baseline. Significant changes in micronutrient intake are included in Table 3. There was a significant increase in vitamin C (*p* < 0.001) and E (*p* = 0.037) intakes (Table 3). There was a significantly higher intake of folate at week 12 compared to baseline (*p* = 0.012) and when investigated further this was attributed to folate naturally occurring in foods (*p* = 0.002).

At baseline the amount of fruit eaten (fresh and total) is the same whether or not kiwifruit is excluded, as participants were instructed not to eat any kiwifruit during the lead-in phase of the trial, during which time the baseline food diaries were completed (Table 3). Analysis of total and fresh fruit intake including and excluding kiwifruit consumption showed that participants were substituting some of their usual fruit intake for kiwifruit during the study (Table 3). At week 12 compliance with the intervention was calculated from the food diary data to be 91%. This was calculated for every individual as the percentage of the six kiwifruit consumed over three days.

### 3.2. Dietary Vitamin C Intakes

**Figure 2:**
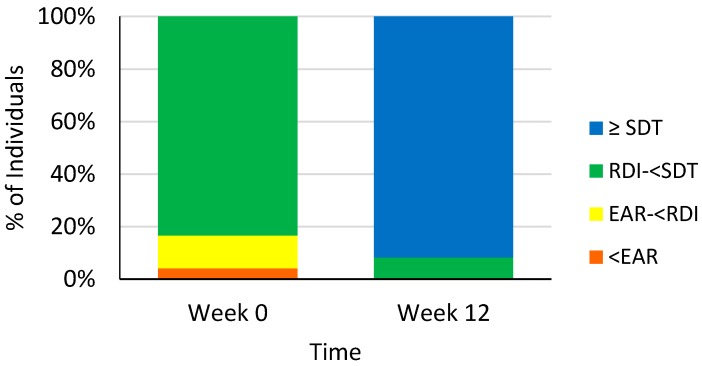
Dietary vitamin C intake of individuals at weeks 0 and week 12 meeting the estimated average requirement (EAR) (30 mg/day), recommended dietary intake (RDI) (45 mg/day) and suggested dietary target (SDT) to reduce chronic disease risk (220 mg/day for men and 190 mg/day for women) [36].

At baseline the majority of participants (*n* = 20) met the New Zealand RDI for vitamin C of 45 mg/day (Figure 2). There was only one participant who had a dietary intake below the New Zealand Estimated Average Requirement (EAR) (30 mg/day) and there were no participants reaching the New Zealand Ministry of Health suggested dietary target (SDT) to reduce chronic disease risk, that is, 220 mg/day for men and 190 mg/day for women (Figure 2). At the end of the study the majority of participants (*n* = 22) had dietary intakes reaching the SDT and the other participants had intakes above the Recommended Dietary Intake (RDI) (*n* = 2) (Figure 2).

### 3.3. Anthropometry and Blood Pressure

From baseline to the end of the study there were no significant changes in weight, BMI or fat mass. There was a significant decrease in waist circumference at week 6 of 0.9 cm (*p* = 0.019) and 3.1 cm at week 12 (*p* = 0.001) compared to baseline (Table 4). Although waist-to-hip ratio was significantly lower at week 12 compared to baseline (*p* = 0.032), the difference was small and not clinically significant (Table 4). Blood pressure also decreased over the duration of the study (Table 3). At week 6 diastolic and systolic blood pressure had decreased from baseline by 3 mmHg *(p* = 0.040) and 5 mmHg (*p* = 0.026) respectively (Table 4). At week 12 systolic and diastolic blood pressure were 4 mmHg (*p* = 0.029) and 6 mmHg (*p* = 0.003) lower than baseline (Table 4). At baseline 42% (*n* = 11) of participants recorded taking medication that lowers blood pressure. There were changes to blood pressure medication for only two of these participants during the trial. One participant had their blood pressure medication dose reduced by half from week 6 to 12 and another participant had it increased between baseline and week 6 but then reduced back to the baseline dose between week 6 and 12.

### 3.4. Biochemical Indices

There was a small but statistically significant decrease in HbA1c of 1 mmol/mol at week 12 compared to baseline (*p* = 0.005) (Table 5). This decrease, however, would not be considered clinically significant. Although fasting glucose significantly increased by 0.2 mmol/L at week 6 (*p* = 0.001), it was only 0.1 mmol/L higher than baseline at week 12 (*p* = 0.046), which is not clinically significant (Table 5).

### 3.5. Plasma Vitamin C Levels

**Figure 3:**
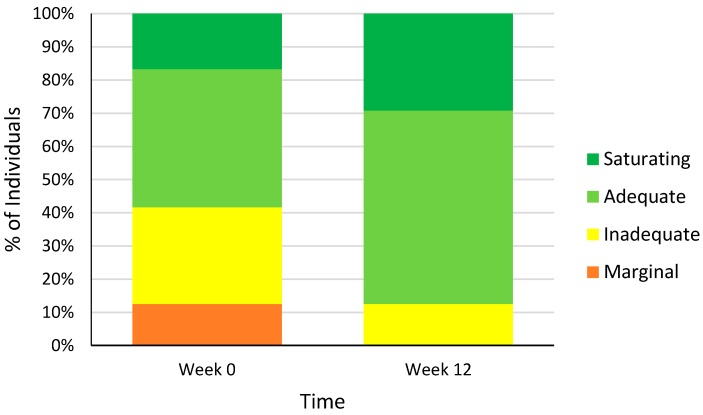
Plasma vitamin C status of individuals at weeks 0 and 12 classified as having saturating (≥70 µmol/L), adequate (50–69 µmol/L), inadequate (24–49 µmol/L) and marginal (11–23 µmol/L) plasma vitamin C. There were no participants classified as having deficient (<11 µmol/L) plasma vitamin C concentrations [38].

At baseline, none of the participants met the criteria for plasma vitamin C deficiency (<11 µmol/L). There were three participants (13%) with marginal (11–23 µmol/L) and seven (29%) with inadequate (24–49 µmol/L) plasma vitamin C concentrations (Figure 3). Over half of the cohort (*n* = 14) had either adequate (42%, 50–69 µmol/L) or saturating (17%, ≥70 µmol/L) plasma vitamin C concentrations at baseline (Figure 3). Consistent with a previous observational study [37], baseline fasting glucose (*r* = −0.450, *p* = 0.024), insulin (*r* = −0.520, *p* = 0.008) and waist circumference (*r* = −0.399, *p* = 0.048) were inversely associated with plasma vitamin C concentrations.

After 12 weeks supplementation, plasma vitamin C concentrations were significantly higher compared to baseline (mean increase of 14 µmol/L, *p* < 0.001) indicating compliance with kiwifruit consumption (Table 5). The increase in plasma vitamin C concentrations was greatest in those who had started the trial with inadequate plasma vitamin C concentration (increase of 22 µmol/L, *p* = 0.004), compared to those who already had adequate plasma vitamin C concentrations at baseline (increase of 7 µmol/L, *p* = 0.029). At the end of the study, there were no participants with marginal plasma vitamin C and only three participants (13%) had inadequate plasma vitamin C concentrations. Fourteen (58%) of the participants had adequate and 7 (29%) of the participants had saturating plasma vitamin C concentrations at week 12 (Figure 3).

### 3.6. Faecal Microbiota

The faecal samples of four participants (one of whom did not complete the trial) were excluded from analysis due to starting a course of antibiotics during the trial. For this analysis, there were 22 participants at week 6 and 21 participants at week 12, due to another participant not completing the trial.

All DNA samples passed quality check and each sample was amplified and barcoded twice to reduce the possibility of losing a sample to sequence failure. Only a single amplification product failed to sequence (1/152 = 0.7%; well within accepted rate of 4%), however the duplicate reaction from this sample sequenced well. Thus, all submitted samples generated good sequence data. A total of 5,527,067 sequences were available for further analysis following quality checking procedures. Each sample generated an average of 72,725 sequences (range 39,216–117,799).

**Figure 4:**
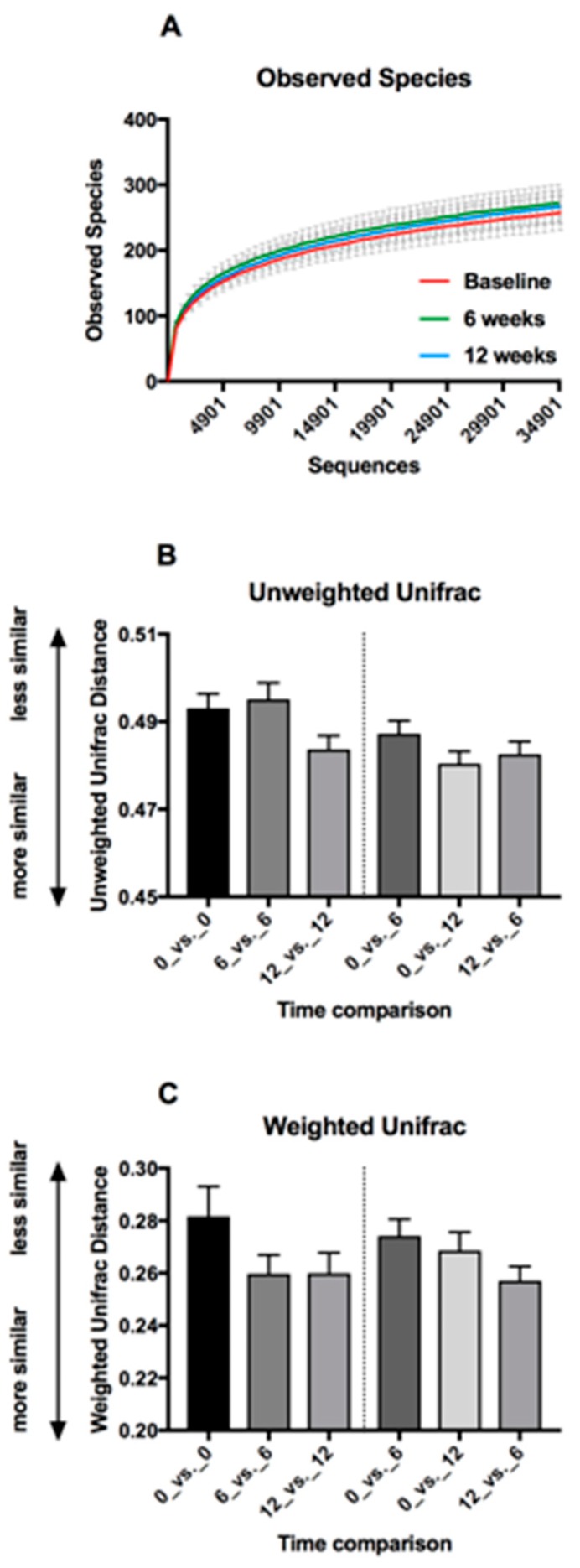
Faecal microbiota diversity metrics. (**A**) Rarefaction curves based on Observed Species metric of alpha diversity, showing coverage of DNA sequences obtained from faecal DNA. Means and 95% CI are shown. (**B**,**C**) Beta diversity metrics comparing microbiota similarity distances within and between groups. Means and SEM are shown. (**B**) Unweighted Unifrac (kinds of bacteria and phylogenetic relatedness). (**C**) Weighted Unifrac (kinds of bacteria, phylogenetic relatedness and relative abundances).

Rarefaction curves showed good coverage of microbiota composition (Figure 4A). Comparison of study time points at a rarefaction level of 35,000 sequences per sample did not show a statistically significant difference in alpha diversity (the complexity in composition of the microbiota) using a variety of metrics (Table 6).

Beta-diversity (unique fraction metric (Unifrac) weighted and unweighted) was used to compare similarities in microbiota composition over time. A comparison of distances within and between time points did not reveal significant changes in community structure with time (Figure 4B,C). Unweighted unifrac distances (kinds of bacteria and phylogenetic relationships) indicated that the microbiotas of the participants tended to be more similar to each other after 12 weeks intervention than at baseline or 6 weeks (Figure 4B). Applying a weighting (based on the relative abundance of OTUs) suggested that the microbiota of participants are more similar to each other at both 6 and 12 weeks than at baseline (Figure 4C).

**Figure 5:**
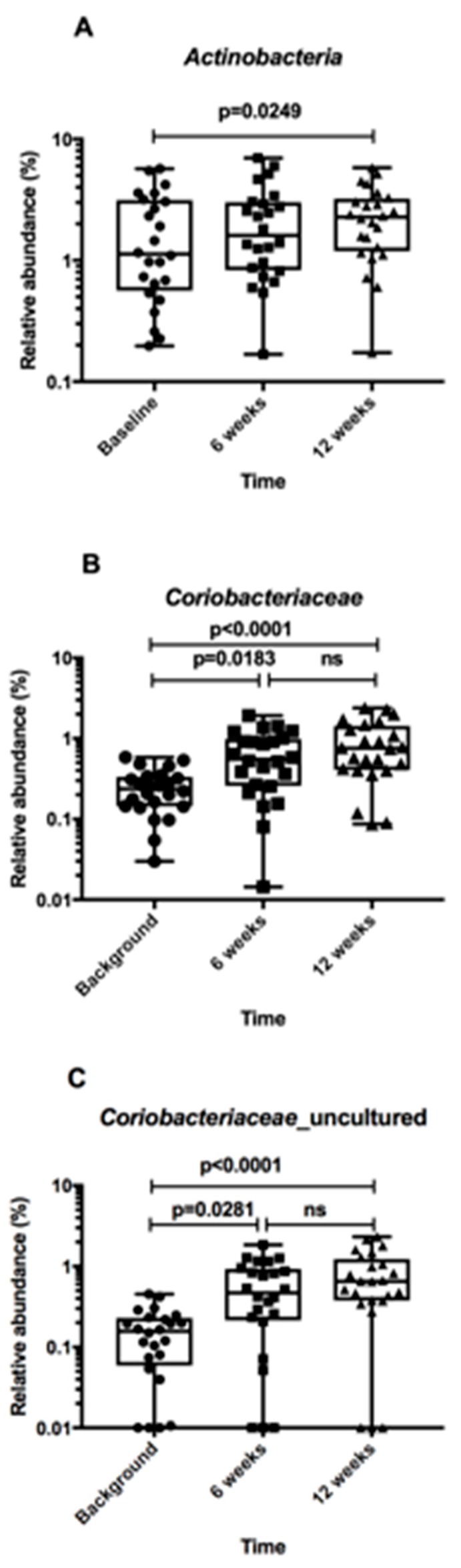
Relative abundances of bacterial groups in faeces of participants at baseline and during supplementation of the diet with kiwifruit. Box and whiskers plots showing individual values and means as horizontal lines. (**A**) Phylum *Actinobacteria*. (**B**) Family *Coriobacteriaceae*. (**C**) OTUs of uncharacterized bacteria of the Family *Coriobacteriaceae*. Statistical evaluation by Wilcoxon matched pairs test using Prism 7.

The complete results of taxonomic analysis of the faecal microbiota at the Family level are supplied in Supplementary Material Table S1. Sequences from members of the bacterial phylum *Actinobacteria* and a bacterial family within this taxon, *Coriobacteriaceae*, were more abundant in faecal samples collected during the kiwifruit intervention compared to baseline (Figure 5A,B). The original OTU table contained 10 clusters identified as *Coriobacteriacae*, including one OTU each in the genera; *Atopobium*, *Collinsella*, *Eggerthella*, *Gordonibacter* and *Senegalimassilia*. The other five OTUs were ‘unidentified’ (i.e., never cultured and characterised). Combining the abundances from these OTUs replicated the significant increase in relative abundance of the Family *Coriobacteriaceae* observed with time (Figure 5C). Thus, it appears that as yet uncultivated member(s) of the *Coriobacteriaceae* contributed to a significant increase in relative abundance during the intervention.

Dietary intervention of two SunGold kiwifruit per day resulted in significantly greater faecal water content at weeks 6 (76%, *p* < 0.001) and 12 (74%, *p* = 0.01) compared to baseline (68%).

## 4. Discussion

In this study, consuming two SunGold kiwifruit per day was associated with a significant increase in plasma vitamin C and fasting glucose, and a decrease in HbA1c, however, these latter two changes were small and, although statistically significant, were not clinically significant. There was a significant reduction in both diastolic and systolic blood pressure. A significant reduction in waist circumference and waist-to-hip ratio was also seen, despite small and non-significant reductions in average BMI and fat mass.

Consumption of two SunGold kiwifruit per day (total of 190 g flesh) for three months provided approximately 300 mg of vitamin C daily from the kiwifruit alone. This increase in dietary vitamin C from the SunGold kiwifruit resulted in most participants meeting the New Zealand Ministry of Health’s suggested dietary target to reduce chronic disease risk [35]. This increase in dietary vitamin C resulted in a significant increase in plasma vitamin C concentrations (mean increase of 14 µmol/L). The increase in plasma vitamin C concentrations was greatest in those who had started the trial with inadequate plasma vitamin C concentration (i.e., <50 µmol/L), compared to those who already had adequate plasma vitamin C concentrations at baseline. This result is consistent with findings from an earlier kiwifruit trial where those with lower baseline plasma vitamin C concentrations showed an earlier and greater response to supplementation, compared to those with adequate plasma vitamin C concentrations at baseline, who showed little effect from supplementation [17]. Three participants (13%) had only adequate plasma vitamin C concentrations at week 12 which may indicate non-compliance or be related to some lifestyle factor, such as smoking, or chronic disease [39].

While all participants met the ADA criteria for prediabetes using HbA1c (39–46 mmol/mol) at baseline, there was still a range of anthropometric and laboratory measures for participants. For example, fasting glucose ranged from 3.9 to 7.5 mmol/L, insulin ranged from 15 to 220 pmol/L and waist circumference ranged from 70 to 125 cm. Fasting glucose, insulin and waist circumference were all inversely associated with plasma vitamin C concentrations at baseline. This result is consistent with previous research [6,7,8,9,10,37,38].

It has been suggested that vitamin C concentrations at baseline may represent a critical factor in predicting the metabolic responses to nutritional interventions tackling oxidative stress and may in part explain why there have been inconsistent findings in RCTs investigating supplementation with vitamin C and glycaemic control [17,39]. Individuals in this study were supplemented with vitamin C-rich kiwifruit; however, there were only three participants with marginal vitamin C levels and no participants with deficient levels at baseline. This may explain why no clinically significant differences in metabolic markers were observed between baseline and week 12. Furthermore, the length of the intervention may need to be longer than 12 weeks to see biological changes [14].

There was a small statistically significant reduction in both diastolic and systolic blood pressure over the duration of the study. This result is unlikely to be due to changes in blood pressure medication as only two participants reported changes to the dose of their blood pressure medication during the study. One of these participants actually had their blood pressure medication dose reduced by half between week 6 and 12 and the other participant was taking the same dose at week 12 as they were at baseline despite having it increased earlier in the study. Those with inadequate vitamin C levels at baseline (i.e., <50 µmol/L) had a greater reduction in blood pressure (decrease of 7 ± 9 mmHg diastolic and 10 ± 11 mmHg systolic) compared to those who had adequate levels (decrease of 2 ± 8 mmHg diastolic and 5 ± 10 mmHg systolic) when week 12 was compared to baseline. This may be related to their level of oxidative stress as hypertension has been related to increased oxidative stress and reduced antioxidant status [40]. Kiwifruit contain a wide range of natural antioxidants; they are rich in vitamin C and also contain vitamin E, polyphenols and flavonoids which are all potent antioxidants [41]. This result is consistent with a RCT of smokers, who are also known to have lower vitamin C concentrations, which showed three kiwifruit per day for eight weeks was associated with reductions of 10 mmHg in systolic blood pressure and 9 mmHg in diastolic blood pressure [42].

Despite the small increase in fruit intake at week 12, there were no significant differences in energy, fibre, protein, total fat and total carbohydrate intake when dietary data at week 12 was compared to baseline. This result suggests participants did not make major changes to their diet during the trial. The addition of the SunGold kiwifruit also had no significant impact on their macronutrient intake as it is relatively low in calories (63 kcal/100 g), protein (1.0 g/100 g), fat (0.3 g/100 g) and carbohydrate (16 g/100 g) [16]. However, SunGold kiwifruit does contain 12 g/100 g of sugar [16], which may have contributed to the significantly higher intake of fructose (increase of 6 g) and glucose (increase of 6 g) at week 12 compared to baseline. SunGold kiwifruit also contains vitamin E (1.4 mg/100 g) and folate (31 µg/100 g), so would have contributed to the higher intake of these vitamins at week 12 compared to baseline.

Although faecal microbiota diversity was stable across the 12-week intervention when viewing the cohort as a whole, *Coriobacteriaceae* family members showed a significant increase in relative abundance during the course of the study. The assignment of these bacteria to uncharacterised bacterial species was particularly interesting because it suggests that as yet uncultivated bacteria that use substrates associated with kiwifruit (such as pectins) exist in the human faecal microbiota. Future work should include efforts to cultivate these bacteria and to determine their functional characteristics. The *Coriobacteriaceae* are in general a poorly studied bacterial family, however they can chemically transform plant polyphenols [43] and thus their activities may be of interest in promoting human health [44]. Certainly, future work should investigate the potential link between *Coriobacteriaceae* and polyphenolic compounds in the faeces of kiwifruit-fed subjects and chemical derivatives of these substances in the blood circulation. While the increased relative abundances of *Coriobacteriaceae* were small, this does not preclude an important role for the bacteria in bowel ecology because low abundance bacteria in microbial communities can have a disproportionally large effect relative to abundance [45].

Our human intervention trial did not confirm the reported in vitro effect of kiwifruit fermentation [25]. In the vitro study, *Bacteroides* spp., *Parabacteroides* spp. and *Bifidobacterium* spp. were reported to increase in cultures containing kiwifruit and inoculated with human faeces. Increased *Coriobacteriaceae* relative abundances were the only impact of dietary modification seen in the participants in our study.

Dietary intervention in the form of two gold kiwifruit per day led to an increase in stool water content at both 6 and 12 weeks. This indicates a shortened gut transit time (laxative effect) and has been linked to decreases in community diversity in other studies. Stool consistency is strongly associated with gut microbiota richness and composition, enterotypes and bacteria growth rates [46]. In our study, the observation may be confounded by the fact that kiwifruit contains 1.4 g/100 g of fibre [16], which is comprised of both soluble and insoluble components at a ratio of approximately 1:3 [19]. The soluble fibre is made up almost exclusively of pectic polysaccharides that have the ability to retain water and form gels, which increases the size and softness of the faeces and aids stimulation of peristaltic movements [19]. The soluble fibre may be the cause of the increase in faecal water seen in this study. Results from several clinical trials also show that kiwifruit promotes laxation [47,48,49,50].

This was the first study to measure changes in the gut microbiota and vitamin C status associated with a whole fruit (SunGold kiwifruit) in people with prediabetes. The participants were free-living representing a realistic intervention for the general population. The use of a whole food rather than an extract/supplement in this study also makes it more affordable and available for people. Although there was no control group in this study, participants acted as their own control with baseline measurements for comparison and dietary intake was consistent over time. In this study plasma vitamin C status corroborated the food diaries as an objective measure of compliance. The study duration of three months enabled the use of HbA1c as a measure of glycaemic control and allowed us to investigate temporary and longer-term shifts in the microbiota.

As this was a pilot exploratory study with a limited number of participants it may not have been sufficient to identify some clinically relevant associations. Additionally, we did not pre-screen participants for vitamin C intake or status and, as a result, half the study cohort already had adequate plasma vitamin C status at baseline. Furthermore, because we did not include an arm with vitamin C supplementation alone, it is difficult to ascertain the contribution of the vitamin C content of the fruit to the observed effects. When assessing dietary intakes there are also limitations around misreporting and a potential bias in recording “good/bad” foods. 

## 5. Conclusions

Supplementation with two SunGold kiwifruit per day significantly increased plasma vitamin C concentrations and provided small improvements in several markers of metabolic and cardiovascular health. The results of faecal microbiota analysis point to the need for novel bacteriological investigations of *Coriobacteriaceae* in order to explain their faecal abundances and to investigate mechanistic relationships with regard to kiwifruit polysaccharides and polyphenols. Due to the small sample size of this pilot study a larger study is indicated, particularly focusing on recruiting participants with low vitamin C status at baseline and determinations of polyphenol derivatives in faeces and blood.

## Figures and Tables

**Table 1:** Information and samples collected at each study visit.

	Lead-In Phase	Week 0 (Baseline)	Week 6 (Study Mid-Point)	Week 12 (Study Completion)
Questionnaires	Food diary	Demography Medical history Medications Supplements Anthropometry Physical activity	Changes to medications and supplements Anthropometry	Changes to medications and supplements Anthropometry Physical activity Food diary
Blood Tests		Fasting glucose vitamin C HbA1c Lipids Hormones hs-CRP	Fasting glucose	Fasting glucose vitamin C HbA1c Lipids Hormones hs-CRP

**Table 2:** General characteristics of study participants.

Characteristics	*n* = 26
Age (years) (mean ± SD)	66 ± 9
Gender	
Female % (*n*)	50 (13)
Male % (*n*)	50 (13)
Ethnicity	
European % (*n*)	81 (21)
Māori % (*n*)	8 (2)
Samoan % (*n*)	4 (1)
Asian/Chinese % (*n*)	4 (1)
Other % (*n*)	4 (1)
Qualification	
No qualification % (*n*)	27 (7)
Secondary school % (*n*)	19 (5)
Post-secondary certificate, diploma or trade diploma % (*n*)	42 (11)
University % (*n*)	12 (3)
Smoking Status	
Current smoker % (*n*)	15 (4)
Ex-smoker % (*n*)	39 (10)
Non-smoker % (*n*)	46 (12)
Alcohol Status	
Current drinker % (*n*)	73 (19)
Ex-drinker % (*n*)	12 (3)
Non-drinker % (*n*)	15 (4)

**Table 3:** Dietary Intake of participants at week 0 and week 12.

Daily Dietary Intake	Week 0 (*n* = 26)	Week 12 (*n* = 24)
Macronutrients		
Energy (KJ)	7407 ± 2759	7176 ± 1683
Fibre (g)	23 ± 8	23 ± 8
Protein (g)	81 ± 24	80 ± 22
Protein (% of energy)	19 ± 4	19 ± 4
Total fat (g)	72 ± 35	68 ± 18
Total fat (% of energy)	35 ± 6	36 ± 6
Total carbohydrate (g)	190 ± 71	184 ± 49
Total carbohydrate (% of energy)	43 ± 5	43 ± 6
Total available sugars (g)	82 ± 33	86 ± 23
Fructose (g)	16 ± 8	22 ± 6 ***
Glucose (g)	14 ± 6	20 ± 5 ***
Sucrose (g)	35 ± 25	29 ± 16 *
Lactose (g)	14 ± 8	20 ± 5
Maltose (g)	3.0 ± 1.8	2.8 ± 1.5
Total starch (g)	108 ± 43	97 ± 34
Micronutrients †		
vitamin C (mg)	79 ± 35	347 ± 70 ***
vitamin E (mg)	8.5 ± 3.9	10.3 ± 3.3 *
Total folate (µg)	271 ± 126	337 ± 119 *
Folate (naturally occurring) (µg)	236 ± 105	291 ± 98 **
Food Groups		
Fresh fruit including kiwifruit (g)	131 ± 91	237 ± 68 ***
Fresh fruit excluding kiwifruit (g)	131 ± 91	72 ± 55 ***
Total fruit including kiwifruit (g)	184 ± 101	274 ± 104 **
Total fruit excluding kiwifruit (g)	184 ± 101	108 ± 94 **

Values presented as mean ± SD. Paired sample t-tests were used to compare dietary data between times. * Significant at the 0.05 level, ** significant at the 0.01 level, *** significant at the 0.001 level. † Micronutrients that were significantly different when week 12 concentrations were compared to baseline.

**Table 4:** Anthropometric, blood pressure and physical activity measures.

Characteristics	Week 0 (*n* = 26)	Week 6 (*n* = 26)	Week 12 (*n* = 24)
Anthropometry			
Weight (kg)	80.2 ± 19.8	80.1 ± 20.0	77.9 ± 18.6
BMI (kg/m^2^)	29.4 ± 7.3	29.4 ± 7.4	28.6 ± 7.0
Fat mass (%)	34.3 ± 6.6	34.4 ± 6.6	34.0 ± 6.9
Waist circumference (cm)	98.6 ± 15.3	97.7 ± 15.2 *	95.5 ± 14.6 ***
Waist-to-hip ratio	0.90 ± 0.09	0.90 ± 0.09	0.89 ± 0.09 *
Blood Pressure			
Diastolic (mmHg)	76 ± 8	73 ± 9 *	72 ± 10 *
Systolic (mmHg)	129 ± 14	124 ± 17 *	123 ± 18 **
Physical Activity			
(met-minutes/week)	3598 ± 5273		

Values presented as mean ± SD. Wilcoxon Signed Ranks tests were used to compare physical activity between times. Paired sample t-tests were used for all other variables. * Significant at the 0.05 level, ** significant at the 0.01 level, *** significant at the 0.001 level. In addition to the missing data for the two participants who did not complete week 12 there were missing data at baseline and week 6 for fat mass (one participant) and for physical activity at week 12 (one participant).

**Table 5:** Laboratory measures of participants at week 0 and week 12.

Biochemical Indices	Week 0 (*n* = 26)	Week 12 (*n* = 24)
HbA1c (mmol/mol)	43 ± 2	42 ± 2 **
Fasting Glucose (mmol/L)	5.4 ± 0.7	5.5 ± 0.8 *
Plasma vitamin C (µmol/L)	50 ± 19	64 ± 13 ***
Total cholesterol (mmol/L)	5.2 ± 1.3	5.1 ± 1.3
HDL cholesterol (mmol/L)	1.35 ± 0.23	1.35 ± 0.23
LDL cholesterol (mmol/L)	3.3 ± 1.0	3.3 ± 1.1
Triglycerides (mmol/L)	1.1 ± 0.5	1.1 ± 0.4
Cholesterol (total/HDL) ratio	3.9 ± 0.9	3.8 ± 0.9
hs-CRP (mg/L)	1.8 (0.6–3.2)	0.9 (0.5–2.2)
Insulin (pmol/L)	51 (31–73)	42 (31–67)
Ghrelin (pmol/L)	162 (110–204)	154 (119–206)
Leptin (ng/mL)	38 (27–71)	35 (25–75)
Adiponectin (µg/mL)	9 (6–11)	10 (7–12)

Values presented as mean ± SD or median and interquartile range (25th to 75th percentiles). Wilcoxon Signed Ranks tests were used to compare hs-CRP and hormones (insulin, ghrelin, leptin and adiponectin) between times. Paired sample *t*-tests were used for all other variables. * Significant at the 0.05 level, ** significant at the 0.01 level, *** significant at the 0.001 level. In addition to the two participants who did not complete the trial there was missing data at baseline and week 6 for fasting glucose (one participant), plasma vitamin C (one participant), and insulin at week 12 (one participant).

**Table 6:** Diversity (at 35,000 sequences/sample) of participants at week 0, 6 and 12.

Diversity Measure	Week 0 (*n* = 22)	Week 6 (*n* = 22)	Week 12 (*n* = 21)
Observed species	250 (213–316)	279 (232–306)	241 (217–283)
Whole tree (PD)	19 (17–23)	21 (18–22)	20 (17–22)
Shannon index	5.1 (4.6–5.4)	5.4 (4.7–5.8)	5.0 (4.9–5.3)
Simpson’s diversity	0.95 (0.91–0.96)	0.96 (0.93–0.97)	0.94 (0.93–0.95)
Chao index	318 (257–377)	367 (288–396)	338 (307–389)

Values presented as median and interquartile range (25th to 75th percentiles). Wilcoxon Signed Ranks tests were used to compare the diversity between times. There were no significant differences.

## Supplementary Materials

The following is available online at http://www.mdpi.com/2072-6643/10/7/895/s1. Supplementary Table S1. OTU table collapsed to family level, showing number of curated sequences 1 per family per sample.
