# Supplementary material for: SunGold Kiwifruit Supplementation of Individuals with Prediabetes Alters Gut Microbiota and Improves Vitamin C Status, Anthropometric and Clinical Markers
Source: Nutrients. 2018 Jul 12;10(7):895. doi: 10.3390/nu10070895 (PMC6073280; doi:10.3390/nu10070895)
Supplement: Supplementary file 1 [file nutrients-10-00895-s001.pdf]

Supplementary Table 1. OTU table collapsed to family level, showing number of curated sequences per family per sample.

Individual	401	402	403	404	405	406	407	408	409	410	411	412	413	414	415	416	417	418	419	420	421	422	423	425	426	427
Time (weeks)	0	0	0	0	0	0	0	0	0	0	0	0	0	0	0	0	0	0	0	0	0	0	0	0	0	0
**Family**																										
Methanobacteriaceae	0	24	20	0	2	0	2	8	0	0	7	0	0	0	0	0	0	0	1	0	0	10	8	12	0	0
Actinomycetaceae	1	8	0	1	1	0	0	1	20	0	2	2	0	0	0	0	1	1	0	0	0	2	5	0	2	3
Bifidobacteriaceae	461	365	3	150	1031	2978	251	853	2	2463	1981	1771	339	1392	1020	533	46	2595	72	102	2266	354	1811	2978	1756	239
Corynebacteriaceae	0	13	0	0	3	1	0	1	1	2	3	0	1	2	1	1	0	0	0	2	1	2	3	0	1	1
Coriobacteriaceae	94	167	378	96	109	464	33	273	238	176	172	258	167	113	124	49	46	41	213	33	178	136	409	125	108	225
Bacteroidaceae	535	894	1853	20744	8435	1052	22671	5259	1156	2570	1051	724	3517	1695	1905	702	4644	3785	320	2587	6859	2604	3087	1401	1355	1031
Bacteroidales S24-7 group	2	11	0	0	0	0	0	6	0	0	3	3	120	0	2	0	2	0	152	0	0	132	13	36	0	1
Porphyromonadaceae	74	38	128	2857	3616	488	18	854	59	338	515	224	206	134	124	476	3	147	10	284	403	286	0	235	284	264
Prevotellaceae	810	6	0	3	0	200	0	110	0	0	1	8	23	18	67	0	5	6	11	0	0	25	0	0	69	7
Rikenellaceae	61	147	663	540	976	355	805	1299	172	322	505	95	547	39	147	6	164	262	52	169	364	502	338	345	129	568
Bacteroidales	0	0	0	5	0	0	0	0	0	0	0	0	37	0	0	0	0	0	0	0	0	0	0	0	0	0
Flavobacteriaceae	0	0	0	0	0	0	0	14	0	0	0	0	2	0	0	0	0	0	0	0	0	0	0	0	0	0
Gastranaerophilales	0	0	22	0	0	0	0	0	0	0	0	2	12	0	1	0	0	0	0	0	0	0	0	0	0	0
Bacillales_Family XI	2	1	0	1	0	0	0	1	46	0	0	0	0	6	2	2	2	2	3	1	7	3	4	1	28	12
Staphylococcaceae	0	0	0	0	0	0	0	0	0	1	1	0	0	0	0	0	0	0	0	0	0	0	0	0	0	0
Enterococcaceae	0	0	0	316	0	1	0	1	0	0	1	0	0	25	2	1	0	2	0	1	911	2	2	0	2	261
Lactobacillaceae	4	6	0	0	0	2	0	1	0	27	10	13	0	43	110	1	0	27	0	3	2	57	556	6917	375	103
Leuconostocaceae	0	0	0	0	0	5	0	0	0	4	0	0	1	1	1	1	0	1	1	0	0	0	0	0	7	0
Streptococcaceae	202	86	22	71	32	28	24	2	572	63	61	225	12	110	46	55	63	54	3	47	348	18	1760	8	82	328
Caldicoprobacteraceae	0	0	2	0	0	1	1	0	0	0	0	0	0	0	0	0	0	0	0	0	0	1	0	0	0	2
Christensenellaceae	1478	1500	3023	280	344	563	1674	8228	36	130	1204	326	5138	2	883	2	152	9	380	8754	324	1654	359	780	388	1743
Clostridiaceae 1	9	278	90	1932	859	346	14	4633	1	37	3226	5	7	7094	30	1745	268	3	4	37	187	1650	432	2	2265	6324
Clostridiales vadinBB60 group	0	114	28	5	17	0	7	15	17	3	38	1	49	0	2	0	1	0	0	0	0	9	9	32	0	10
Defluviitaleaceae	4	9	237	4	17	21	19	73	0	0	35	4	4	0	2	0	0	0	1	2	1	13	44	16	0	57
Eubacteriaceae	0	35	11	10	0	0	11	0	178	2	3	0	3	0	1	0	1	0	1	5	0	7	1	0	0	2
Clostridiales_Family XI	0	33	0	1	0	0	0	0	22	0	0	0	3	3	1	0	0	4	0	1	0	0	1	1	0	2
Clostridiales_Family XIII	14	2050	136	69	54	131	100	294	271	105	785	59	215	3	37	0	87	962	51	66	49	410	277	108	384	80
Lachnospiraceae	32549	51343	48935	15729	31931	61059	49446	35342	58008	39993	7623	55606	13767	51079	26829	17030	29376	16061	43952	33538	33930	23907	41697	31463	34619	27815
Peptococcaceae	0	12	56	5	3	2	35	34	17	14	62	0	8	0	2	0	3	0	154	1	0	191	24	15	3	1
Peptostreptococcaceae	414	2757	204	2068	6374	2654	582	2076	5	942	6784	708	341	7902	360	10636	8	101	7	8	4212	5582	97	190	752	7742
Ruminococcaceae	14179	14449	12663	7958	21439	17686	29932	34509	5039	13501	8123	9477	13213	5676	14541	14079	8312	8373	8725	12064	15481	22738	13859	10578	11738	20243
Thermoanaerobacteraceae	0	0	1	0	2	0	0	0	0	0	3	0	0	0	0	0	0	0	0	0	0	1	0	0	0	0
Erysipelotrichaceae	3074	6118	1580	2376	2148	8274	605	6452	2499	1884	1976	6296	1301	3184	2626	3961	3619	5230	6036	356	2010	3164	4081	653	5923	5342
Acidaminococcaceae	0	9	0	128	187	0	0	12	137	66	0	220	213	136	0	0	221	4	2	1	4	0	263	79	102	5
Veillonellaceae	2057	37	348	59	828	10	2521	721	2140	30	20	130	27	121	3	820	79	16	4	33	355	2567	17	252	460	120
Fusobacteriaceae	0	0	0	1	0	0	0	2	0	0	2	0	0	0	0	1	0	0	0	2	0	0	0	0	0	1
Leptotrichiaceae	0	0	0	0	0	0	0	0	0	0	0	0	0	0	0	0	0	0	0	0	0	0	0	0	0	0
Victivallaceae	0	0	0	0	0	0	0	0	0	0	0	0	1	0	0	0	0	0	0	0	0	0	0	0	0	0
Victivallales_vadinBE97	0	0	0	0	0	0	0	0	0	0	0	0	0	0	0	0	0	0	0	0	0	1	0	0	0	0
Rhizobiaceae	0	0	0	0	0	0	0	0	0	0	0	0	0	0	0	0	0	0	0	0	0	0	0	0	0	2
Rhodospirillaceae	0	12	0	0	0	0	0	18	0	0	0	0	2	0	1	0	0	0	5	0	0	0	0	0	0	0
Alcaligenaceae	0	3	3	0	17	0	0	1	5	0	4	3	3	0	4	0	0	0	2	0	17	4	3	1	0	1
Oxalobacteraceae	0	1	0	0	0	0	0	0	0	0	2	0	0	0	0	0	0	2	0	0	0	0	0	0	0	0
Neisseriaceae	0	0	0	0	0	0	0	0	0	0	0	0	0	0	0	0	0	0	0	0	0	0	0	0	0	0
Desulfovibrionaceae	5	32	4	106	87	6	222	142	0	13	62	12	34	3	19	0	42	50	165	13	25	9	175	4	1	17
Campylobacteraceae	0	0	0	0	0	0	0	0	0	0	0	0	0	0	0	0	0	0	0	0	0	0	0	0	0	0
Aeromonadaceae	0	0	0	0	0	0	0	0	0	0	0	0	0	0	0	0	0	0	0	0	0	0	0	0	11	0
Cardiobacteriaceae	0	0	0	0	0	0	0	0	0	0	0	0	0	0	0	0	0	0	0	0	0	0	0	0	0	0
Enterobacteriaceae	3	15	4	235	33	0	1	3	1	9	15	0	51	13	0	1	0	0	0	1948	19	43	3	20	202	271
Pasteurellaceae	82	2	0	5	8	0	409	0	0	0	2	0	0	0	1	189	1	2	0	7	96	18	1	0	212	100
Moraxellaceae	0	0	0	0	1	0	0	0	0	0	0	0	1	0	0	1	0	0	0	0	0	0	0	0	0	0
Pseudomonadaceae	0	0	0	0	0	0	0	0	0	0	0	0	0	0	0	0	0	0	0	0	0	0	0	0	0	0
Spirochaetaceae	0	0	0	0	0	0	2	0	0	0	0	0	0	0	0	0	0	0	0	0	0	0	0	0	0	0
Anaeroplasmataceae	0	0	0	0	0	0	0	0	0	0	0	0	0	0	0	0	0	0	0	0	0	30	0	0	0	0
Mollicutes RF9_uncultured	1192	13	172	63	0	1	131	1278	0	1	89	22	21	1	421	0	133	34	112	0	0	1418	0	337	0	0
Mollicutes_NB1-n	0	6	19	0	0	0	0	0	0	0	0	0	0	0	0	0	0	0	0	0	0	0	0	0	0	0
Opitutae vadinHA64	0	0	0	0	0	0	0	0	0	0	0	0	1	0	0	0	0	0	0	0	0	2	0	0	0	0
Verrucomicrobiaceae	0	11	145	10357	192	0	481	57	1	0	3472	21	13074	3	5	1	1	0	84	561	10	30	690	29	2	3

Individual	401	402	403	404	405	406	407	408	409	410	411	412	413	414	415	416	417	418	419	420	421	422	423	425	426	427
Time (weeks)	6	6	6	6	6	6	6	6	6	6	6	6	6	6	6	6	6	6	6	6	6	6	6	6	6	6
**Family**																										
Methanobacteriaceae	0	7	4	0	1	0	0	1	0	0	3	0	0	0	0	0	0	0	0	0	17	5	5	1	0	3
Actinomycetaceae	2	2	24	1	1	0	0	1	16	2	1	1	0	1	0	0	3	2	1	1	3	0	2	14	0	1
Bifidobacteriaceae	1365	281	1	613	433	3550	56	928	2	4435	1407	2029	1014	769	246	354	371	1547	245	85	3068	279	2117	778	320	1553
Corynebacteriaceae	0	5	1	0	1	2	0	0	0	0	3	0	0	0	0	0	0	1	0	1	4	4	0	3	0	4
Coriobacteriaceae	92	581	764	762	900	320	51	759	129	362	422	360	268	8	196	97	242	75	184	655	955	432	447	746	1632	712
Bacteroidaceae	835	2065	15991	946	9188	1653	15010	2489	4666	5626	1047	889	485	376	2556	1191	18174	10328	11587	12395	6001	8432	3957	705	1435	8877
Bacteroidales S24-7 group	0	35	0	1	2	1	0	6	0	0	1	7	28	0	2	0	15	1	1032	953	0	15	111	0	0	53
Porphyromonadaceae	170	79	110	129	3455	1365	45	505	93	1452	163	192	159	6	227	606	54	623	420	849	500	582	1028	189	312	2
Prevotellaceae	2804	21	0	3	0	1094	0	152	0	0	0	239	13	1	90	0	27	67	184	0	0	135	1	69	4	0
Rikenellaceae	1	262	601	137	1108	178	902	789	254	1131	313	248	204	0	269	152	795	886	291	1354	1252	921	711	70	490	1246
Bacteroidales	0	0	0	9	0	0	0	0	0	0	0	0	4	0	0	0	0	0	0	0	0	0	0	0	0	0
Flavobacteriaceae	0	0	0	0	0	0	0	5	0	0	0	0	16	0	0	0	0	0	0	0	0	0	0	0	0	0
Gastranaerophilales	0	0	13	0	0	0	0	0	0	0	0	0	15	0	2	0	0	0	0	0	0	0	0	0	0	0
Bacillales_Family XI	0	2	0	3	0	1	7	0	8	0	0	2	0	2	1	8	5	3	1	3	1	3	2	1	1	2
Staphylococcaceae	0	0	0	0	0	0	0	0	0	0	0	0	0	0	0	0	1	0	0	0	0	0	0	0	0	0
Enterococcaceae	2	0	0	844	0	0	1	0	2	0	0	0	0	0	2	0	3	0	0	4	662	1	0	1	34	2
Lactobacillaceae	1	3	0	0	0	1	4	0	0	14	69	0	0	2	233	72	0	23	1	12	90	24	574	55	65	321
Leuconostocaceae	0	0	0	3	0	0	0	0	0	0	0	0	0	0	1	1	0	0	1	0	0	0	1	0	0	17
Streptococcaceae	30	124	2	342	5	21	132	0	165	91	119	32	4	204	42	13	135	109	78	32	692	24	13	120	0	1091
Caldicoprobacteraceae	0	1	1	7	2	0	3	1	0	0	0	0	0	0	0	0	0	0	0	0	2	4	1	0	5	4
Christensenellaceae	167	2198	1644	534	3220	262	642	2802	19	169	1810	271	12515	4	1797	5	30	9	197	4659	1778	1901	1011	860	2482	453
Clostridiaceae 1	47	1148	9	11255	1200	201	106	1722	1	56	1717	15	225	273	99	2705	40	3	5	1026	213	1664	141	1559	4635	823
Clostridiales vadinBB60 group	0	209	28	6	2	0	7	6	12	2	1	0	79	0	0	0	0	0	4	10	162	20	35	0	47	28
Defluviitaleaceae	0	5	108	60	29	7	20	2	17	0	5	0	4	0	1	0	0	0	1	8	29	7	15	2	28	133
Eubacteriaceae	0	23	4	10	4	0	3	0	28	0	3	0	4	0	0	0	0	0	4	5	0	2	0	1	7	3
Clostridiales_Family XI	0	4	5	2	0	4	0	0	17	0	0	0	1	1	0	0	1	0	0	1	20	0	1	1	2	0
Clostridiales_Family XIII	6	1119	250	213	80	137	70	225	131	90	582	55	233	1	51	0	270	408	63	118	345	434	81	424	133	189
Lachnospiraceae	34715	57175	29370	27036	38954	46397	27862	18508	23895	33425	25915	43085	48701	46084	32223	34418	66662	24185	34348	31374	23168	35565	12768	25310	38935	30419
Peptococcaceae	0	12	19	29	9	4	29	27	63	15	26	1	10	1	2	0	0	0	116	2	39	281	26	3	0	31
Peptostreptococcaceae	2131	1584	29	9792	4830	1438	312	3220	1	988	8881	171	344	5616	145	3325	1000	20	137	2032	2662	4709	270	471	9187	479
Ruminococcaceae	11989	25513	11834	20637	31506	20581	16278	18259	7266	19551	21778	18311	27922	760	14086	21615	13629	11707	17655	15361	20583	32804	24369	11621	22540	26750
Thermoanaerobacteraceae	0	0	1	0	0	0	3	0	0	0	0	0	0	0	0	0	0	0	1	0	0	10	0	0	0	7
Erysipelotrichaceae	4246	7282	1568	1947	2703	5483	271	2734	2354	1175	1774	2994	3488	1199	1402	2311	12056	8465	1252	1742	3200	4810	576	2481	2342	1885
Acidaminococcaceae	0	32	0	39	352	9	0	0	682	105	1	196	660	10	0	0	153	13	14	23	17	0	36	547	4	397
Veillonellaceae	398	80	425	99	109	143	826	1523	534	36	19	924	58	309	71	978	505	30	10	44	1467	2632	216	231	71	17
Fusobacteriaceae	0	0	4	1	0	0	0	0	0	0	0	0	0	0	0	0	0	2	0	0	8	1	0	0	0	0
Leptotrichiaceae	0	0	0	0	0	0	0	0	0	0	0	0	0	0	0	0	1	0	0	0	0	0	0	0	0	0
Victivallaceae	0	6	0	0	0	0	0	0	0	0	0	0	0	0	0	0	0	0	0	0	0	0	0	0	0	0
Victivallales_vadinBE97	0	0	0	0	0	0	0	0	0	0	0	0	0	0	0	0	0	0	0	0	0	3	0	0	0	0
Rhizobiaceae	0	0	0	0	0	0	0	0	0	0	0	0	0	0	0	0	0	0	0	0	0	0	0	0	0	0
Rhodospirillaceae	0	87	0	0	0	0	0	23	0	0	0	0	1	0	0	0	0	0	0	0	0	0	0	0	0	0
Alcaligenaceae	0	2	9	0	16	1	0	7	11	4	11	2	9	0	5	0	3	3	3	3	6	10	0	0	5	70
Oxalobacteraceae	0	0	1	0	0	0	0	0	0	0	0	0	0	0	0	0	0	1	3	0	7	0	3	0	0	0
Neisseriaceae	0	0	0	0	0	0	0	0	0	0	0	0	0	0	0	0	0	0	0	0	0	1	0	0	0	1
Desulfovibrionaceae	1	70	7	7	84	6	224	32	0	15	22	22	76	0	53	2	35	185	122	12	14	16	14	3	16	428
Campylobacteraceae	0	0	0	0	0	0	0	0	0	0	0	0	0	0	0	0	0	0	0	0	13	0	0	0	0	0
Aeromonadaceae	0	0	0	0	0	0	0	0	0	0	0	0	0	0	0	0	0	0	0	0	0	0	0	1	0	0
Cardiobacteriaceae	0	0	0	0	0	0	0	0	0	0	0	0	0	0	0	0	0	0	0	0	0	0	0	0	0	0
Enterobacteriaceae	0	1	44	60	3	0	1	1	0	10	5	2	8	14	3	3	0	1	0	1091	0	14	6	13824	3	0
Pasteurellaceae	77	20	0	0	54	1	239	0	0	0	90	0	2	2	8	12	11	85	0	0	0	12	0	95	2	1
Moraxellaceae	9	0	0	0	0	0	1	0	0	0	0	1	0	0	1	2	0	0	0	0	0	0	0	0	0	0
Pseudomonadaceae	0	2	0	0	0	0	0	0	0	0	0	0	0	0	0	0	0	0	0	0	0	0	0	0	0	0
Spirochaetaceae	0	0	0	0	0	0	1	0	0	0	0	0	0	0	0	0	0	0	0	0	0	0	0	0	0	0
Anaeroplasmataceae	0	0	0	0	0	0	0	0	0	0	0	0	0	0	0	0	0	0	0	0	0	42	0	0	0	0
Mollicutes RF9_uncultured	58	62	118	33	79	0	29	332	1	0	0	10	517	0	247	0	0	0	3155	1	0	1715	423	0	0	0
Mollicutes_NB1-n	0	6	8	0	0	0	0	0	0	0	0	0	0	0	0	0	0	0	0	0	0	0	0	0	0	0
Opitutae vadinHA64	0	1	0	0	0	0	0	0	0	0	0	0	3	0	0	0	0	0	0	0	0	6	0	0	0	0
Verrucomicrobiaceae	1	19	259	153	52	1	454	227	0	1	2	0	3590	0	6	0	0	0	1186	3180	309	2	513	206	15	2018

Individual	401	402	403	404	405	406	407	408	410	411	412	413	414	415	416	417	419	420	421	422	423	425	426	427
Time (weeks)	12	12	12	12	12	12	12	12	12	12	12	12	12	12	12	12	12	12	12	12	12	12	12	12
**Family**																								
Methanobacteriaceae	0	5	6	0	2	0	0	5	0	0	0	0	0	0	0	0	0	0	10	10	9	0	0	3
Actinomycetaceae	0	0	0	3	3	1	0	0	0	1	0	1	3	0	0	1	2	3	0	3	2	2	2	3
Bifidobacteriaceae	1685	155	2	876	680	1549	37	359	3054	2569	695	563	1458	1097	1403	329	227	17	1092	788	2682	279	571	1273
Corynebacteriaceae	0	5	0	0	1	0	0	0	0	1	0	2	0	4	4	1	1	15	0	0	0	1	1	3
Coriobacteriaceae	298	998	2073	1528	1469	619	82	794	319	572	362	406	62	361	62	730	291	332	469	277	586	392	1307	1047
Bacteroidaceae	1546	575	692	627	10283	588	11887	7233	10009	3030	3561	3452	743	1656	237	2742	10396	3090	4148	2306	2387	1573	327	3639
Bacteroidales S24-7 group	0	1	0	0	0	0	0	2	0	1	13	56	0	1	0	1	2411	912	0	18	32	0	0	23
Porphyromonadaceae	694	67	157	134	3635	195	315	1339	1656	376	767	414	78	141	58	1	168	199	208	366	461	196	87	0
Prevotellaceae	682	0	0	1	1	76	0	240	0	0	117	64	3	89	0	2	53	0	0	308	0	6	0	0
Rikenellaceae	45	192	685	86	2202	198	670	3029	2062	1149	506	1178	31	172	5	191	273	472	1710	931	833	155	123	748
Bacteroidales	0	0	0	1	0	0	0	0	0	0	0	54	0	0	0	0	0	0	0	0	0	0	0	0
Flavobacteriaceae	0	0	0	0	0	0	0	19	0	0	0	41	0	0	0	1	0	0	0	0	0	0	0	0
Gastranaerophilales	0	0	28	0	0	0	0	0	0	0	12	27	0	0	0	0	0	0	0	0	0	0	0	0
Bacillales_Family XI	2	4	0	2	3	0	0	0	0	1	2	0	3	2	8	10	1	5	0	0	3	16	2	6
Staphylococcaceae	1	0	0	1	0	0	0	0	0	0	0	0	0	0	0	0	0	0	2	0	0	1	0	0
Enterococcaceae	1	0	0	367	0	0	0	0	0	44	0	1	0	3	1	0	0	0	64	0	0	153	2	1
Lactobacillaceae	3	10	0	1	0	1	0	0	0	97	0	3	4	115	1	0	1	4	104	85	1182	216	385	205
Leuconostocaceae	1	0	0	1	0	0	0	0	0	0	5	0	0	1	0	0	0	3	0	0	0	0	0	0
Streptococcaceae	141	281	22	283	11	20	34	1	42	100	31	9	72	149	38	64	5	167	76	18	66	278	248	1061
Caldicoprobacteraceae	0	0	0	0	0	0	1	0	0	0	0	2	0	0	0	0	1	0	0	1	0	0	0	2
Christensenellaceae	885	1061	3875	362	1120	269	1175	3239	126	2168	530	11756	2	190	1	11	311	4641	1144	1419	1036	520	918	203
Clostridiaceae 1	78	738	3	15912	984	557	0	1127	383	2191	11	1078	404	39	1038	10	1	1423	268	395	10	5703	2195	8
Clostridiales vadinBB60 group	0	54	23	6	28	3	2	30	8	3	1	428	0	0	0	0	0	3	61	11	8	0	3	3
Defluviitaleaceae	0	3	160	38	37	20	23	12	0	5	8	4	0	1	0	0	1	1	6	10	4	1	14	15
Eubacteriaceae	0	11	6	0	3	0	9	1	1	1	0	4	0	0	0	13	7	2	0	1	1	2	5	2
Clostridiales_Family XI	0	0	1	2	8	1	0	0	4	0	1	26	1	4	0	1	0	1	2	0	0	0	2	0
Clostridiales_Family XIII	20	1396	113	130	45	70	67	128	53	1210	55	367	3	32	3	150	43	119	115	319	69	364	69	181
Lachnospiraceae	42375	32326	61673	18550	28536	21556	32214	22686	22498	26429	65476	34173	48920	35800	32196	70198	39933	25639	21488	27421	32514	17388	30327	40753
Peptococcaceae	1	2	47	13	3	2	20	22	17	41	1	6	0	1	0	0	90	1	17	230	18	0	0	5
Peptostreptococcaceae	973	3435	17	8579	5794	3949	125	1300	899	7748	85	332	5060	1025	9914	662	69	5848	3884	2848	728	756	6918	206
Ruminococcaceae	14965	12334	13504	7674	25016	10364	19856	25146	15446	21522	23256	21478	9948	19189	16874	12074	13851	10102	13427	23222	18577	6436	18558	17472
Thermoanaerobacteraceae	0	0	0	0	4	0	4	1	0	0	0	0	0	0	0	0	0	0	0	3	0	0	0	0
Erysipelotrichaceae	2629	8218	2770	10943	4376	7974	455	3988	1282	3649	5514	1492	1219	2619	8621	7554	3516	965	2248	3525	1038	2464	2715	1527
Acidaminococcaceae	0	4	1	10	238	0	0	23	130	7	514	9	26	0	0	12	14	0	47	0	8	461	1	110
Veillonellaceae	853	13	299	56	511	2	1431	1231	5	36	282	9	402	9	705	186	7	211	31	3483	214	172	13	11
Fusobacteriaceae	0	0	0	0	0	0	0	0	0	0	0	0	0	0	3	0	0	1	0	0	0	0	0	8
Leptotrichiaceae	0	0	0	0	0	0	0	0	0	0	0	0	0	0	0	0	0	2	0	0	0	0	0	0
Victivallaceae	0	1	0	0	0	0	0	0	0	0	0	5	0	0	0	0	0	0	0	0	0	0	0	0
Victivallales_vadinBE97	0	0	0	0	0	0	0	0	0	0	0	0	0	0	0	0	0	0	0	3	0	0	0	0
Rhizobiaceae	0	0	0	0	0	0	0	0	0	0	0	0	0	0	0	0	0	0	0	0	0	0	0	0
Rhodospirillaceae	0	7	0	0	0	0	0	54	0	0	19	4	0	0	0	0	18	0	0	0	0	0	0	0
Alcaligenaceae	0	0	1	0	10	0	0	8	3	47	22	6	0	3	3	1	1	1	12	5	1	0	1	7
Oxalobacteraceae	0	0	2	0	0	0	0	0	0	1	0	1	0	0	0	0	3	0	1	0	3	0	0	0
Neisseriaceae	0	0	0	0	0	0	0	0	0	0	0	0	0	0	0	0	0	0	0	0	0	0	0	0
Desulfovibrionaceae	1	8	4	3	36	5	85	110	3	27	62	36	121	5	1	61	112	2	31	6	35	2	7	358
Campylobacteraceae	0	0	0	0	0	0	0	0	0	0	0	0	0	0	0	0	0	0	0	0	0	0	0	0
Aeromonadaceae	0	0	0	0	0	0	0	0	0	0	0	0	0	0	0	0	0	0	0	0	0	2	0	0
Cardiobacteriaceae	0	3	0	0	0	0	0	0	0	0	0	0	0	0	0	0	0	1	0	0	0	0	0	0
Enterobacteriaceae	0	3	0	16	0	0	0	4	27	27	2	10	56	1	279	1	12	6360	97	61	3	14549	0	0
Pasteurellaceae	12	1	1	0	0	0	82	15	1	165	0	0	2	9	122	14	0	99	3	23	1	370	3	0
Moraxellaceae	0	1	0	0	0	1	0	0	0	1	0	0	0	0	0	0	0	0	0	0	0	0	0	1
Pseudomonadaceae	0	0	0	0	0	0	0	0	0	0	0	0	0	0	0	0	0	0	0	0	0	0	0	0
Spirochaetaceae	0	0	0	0	0	0	0	0	0	0	0	0	0	0	0	0	0	0	0	0	0	0	0	0
Anaeroplasmataceae	0	0	0	0	0	0	0	0	0	0	0	0	0	0	0	0	0	0	0	16	0	0	0	0
Mollicutes RF9_uncultured	2674	2	376	11	10	0	62	378	0	1	18	199	2	1	0	0	338	1	0	954	377	31	2	0
Mollicutes_NB1-n	0	0	28	0	0	0	0	0	0	0	0	0	0	0	0	0	0	0	0	0	0	0	0	0
Opitutae vadinHA64	0	0	0	0	0	0	0	0	0	0	0	3	0	0	0	0	0	0	0	0	0	0	0	0
Verrucomicrobiaceae	0	27	2	517	34	1	4	115	0	0	1	6084	1	6	1	2	380	140	654	0	111	20	14	1692
